# Indian Society of Electrocardiology Position Statement on Improving CPR Awareness in the Indian Public^☆^

**DOI:** 10.1016/j.ihj.2026.05.004

**Published:** 2026-06-10

**Authors:** Aditya Kapoor, Ajit Bhagwat, Ashish Nabar, Satyavir Yadav, B. Hygriv Rao, Yash Lokhandwala, S.B. Gupta, Kamlesh Raut, Ketan K. Mehta, Deepa Kane, Rusheek Rawal, Aniruddha Vyas, Mandar Mahavir Shah, Amit Vora, Akshay Mehta, Joy M. Thomas, Arun David Arul, Nayani Makkar, Narayanan Namboodiri, Shomu Bohora, Deepak Padmanabhan, Kinjal Goyal, Sunil Sathe, Ajit Desai, Vishal Gavali, Jitendra Makkar, Nitish Naik, Ajay Naik, Rakesh Yadav, Anoop Gupta, Debabrata Roy, Jyotirmoy Pal, Mangesh Tiwaskar, Sriniwas Samavedam, Jigeeshu V. Divatia

**Affiliations:** a Sanjay Gandhi Postgraduate Institute of Medical Sciences, Lucknow, India; b Kamalnayan Bajaj Hospital, Aurangabad, India; c Arrhythmia Associates, Mumbai, India; d All India Institute of Medical Sciences, Delhi, India; e Krishna Institute of Medical Sciences Group of Hospitals, Hyderabad, India; f Care Clinic, Mumbai, India; g Max Hospital, Shalimar Bagh, Delhi, India; h Suchak Hospital, Mumbai, India; i Seth GSMC & KEM Hospital, Mumbai, India; j Vishesh Jupiter Hospital, Indore, India; k Tata Main Hospital, Jamshedpur, India; l Dr Mehta's Heart and Physiotherapy Centre, Mumbai, India; m Apollo Specialties Hospital, Chennai, India; n Sri Chitra Thirunal Institute of Medical Sciences and Technology, Trivandrum, India; o Apex Heart Institute, Gandhinagar, India; p Narayana Institute of Cardiac Sciences, Bengaluru, India; q Healthy Mind, Pune, India; r Cardiac Care and Counselling Centre, Pune, India; s Jaslok Hospital, Mumbai, India; t Eternal Hospital, Jaipur, India; u Marengo Asia CIMS Hospital, Ahmedabad, India; v Epic Multispeciality Hospital, Ahmedabad, India; w NH-RTIICS, Kolkata, India; x Sagar Dutta Medical College, Kolkata, India; y Shilpa Medical Research Centre, Mumbai, India; z Sindhu Hospitals, Hyderabad, India; aa Lilavati Hospital, Mumbai, India

## Abstract

Out-of-hospital cardiac arrest (OHCA) is a major public health challenge in India. Cardiopulmonary resuscitation (CPR) awareness, training, and bystander response rates remain critically low across the country.

**Objective:**

This Indian Society of Electrocardiology position statement aims to promote nationwide CPR awareness, strengthen community response to sudden cardiac arrest (SCA) and improve survival in OHCA, with measures adapted to India-specific systems of care and bystander training.

**Main themes:**

The statement highlights the SCA burden, early recognition, rapid response, barriers to community CPR training in India, dispatcher-assisted CPR, and public access defibrillation (PAD). It emphasizes school-based CPR education, corporate social responsibility partnerships, legislative support, and development of a national CPR ecosystem. Role of digital innovations, mobile applications, virtual reality, mobile CPR training units, psychological barriers and the bystander effect are emphasized.

**Conclusion:**

The position statement advocates transforming CPR from a medical skill into a societal responsibility and improve OHCA survival in India.

## 1 Introduction

Sudden cardiac arrest (SCA) is usually defined as sudden cessation of cardiac activity in which the victim becomes unresponsive, with either persisting gasping respirations or absence of any respiratory movements, and no signs of circulation i.e. no perceptible pulse. Sudden Cardiac Death (SCD) is an out-of-hospital, sudden natural death presumed to be of cardiac cause (i.e., without obvious extracardiac cause), occurring with a rapid, witnessed collapse within 1 h of symptoms onset, or within 24 h of last being seen in the usual state of health if unwitnessed. Sudden arrhythmic death syndrome (SADS) is defined as an unexplained sudden death occurring in an individual aged >1 year with negative pathological and toxicological assessment at autopsy.^1^ Cardiac arrest is therefore used to signify an event that can be reversed, usually by cardiopulmonary resuscitation (CPR), defibrillation and/or administration of medications. Global experience shows that survival from OHCA depends critically on two factors—immediate bystander CPR and early defibrillation. Each minute without CPR decreases survival by 7–10%.^2^ Although it can double or triple survival in OHCA; bystander CPR rates in India remain alarmingly low due to lack of awareness. Recognizing this unmet need, the Indian Society of Electrocardiology (ISE) proposes the following expert commandments for CPR awareness tailored for the Indian population. Addressing these issues should be a national priority that seeks to transform CPR from a medical skill into a societal responsibility.

## 2 Burden of SCD

### 2.1 Global context

Although the annual burden of SCD worldwide is difficult to estimate, it accounts for approximately 185-450,000 deaths each year in USA and the corresponding numbers for European Union are reported to vary from 250 to 350,000.

^3^^,^^4^ SCD accounts for nearly half of all cardiovascular deaths, 20% of overall mortality and carries a higher burden of premature mortality than any single cancer in men or women across all age groups.^4^^,^^5^ In about 50% of victims, there is no prior warning, making the event both unpredictable and devastating. The true incidence of SCD is difficult to ascertain due to varied definitions used and different methodologies employed. Retrospective studies relying on death certificates often overestimate SCD rates—sometimes by as much as 200–300%.^6^ More accurate estimations require prospective surveillance using multiple data sources such as first responders, hospitals, autopsy records, and community surveillance. Long-term prospective studies provide critical insights into SCD incidence. In the Framingham Heart Study, which followed 5209 individuals aged 28–62 years over 50 years, SCD accounted for 358 events, representing 6.9% of the cohort.^7^ In the Paris Prospective Study comprising middle-aged policemen, 118 (5·7%) of 2083 deaths were as a result of SCD over a period of 23 years.^8^ The Oregon Sudden Unexpected Death Study was a more comprehensive community-based study conducted in Multnomah County (∼600,000 population) that used multisource surveillance. It reported an annual incidence of SCD as 53 per 100,000 residents, comprising 5.6% of all deaths.^9^ The Maastricht study from Netherlands (1991–1994) reported an annual incidence of OHCA total cardiac arrests of 90–100 per 100,000 in residents aged 20–75 years.^10^ SCD incidence ranges from 50 to 100 per 100,000 in populations of Europe, Australia, and North America, with slightly lower rates in Asia; data from Africa and South America remain limited. Incidence of SCD is higher among Black compared to White populations.^11^ **These statistics are summarized in** Table 1.

**Table 1:** SCD incidence in major studies.

Study	Definition	Source	Estimated Annual Incidence of SCD	Proportion of SCD
The Framingham Heart Study^7^ Circulation 2004	SCD was defined as a death that occurred within 1 h of the onset of symptoms and that had no other probable cause of death suggested by the medical record	Framingham Heart Study cohort initiated in 1948 with 5209 men and women aged 28–62 years; Offspring cohort (children and spouses) enrolled in 1971	NA	SCD accounted for 358 events, representing 6.9% of the cohort
Paris prospective study^8^ Circulation 1999	Natural death occurring within 1hr of onset of Acute symptoms	Multiple source Native Frenchman in Paris Civil Service	NA.	5.6% of total mortality and 19% of CV mortality.
SCD in US (1989-1998) Zheng et al^5^ Circulation 2001	SCD is a death that reported as occurring out of hospital or in the ER or as dead on arrival with underlying cause of death reported as cardiac cause	US vital statistics Death certificate	456,076 SCD over the study period.	63% of cardiac deaths.
Burden of SCD Multiple source surveillance versus retrospective death certificate-based review in a large U.S. community Sumeet. S. Chugh et al^9^ JACC 2004	Sudden unexpected death either witness 1hr of symptoms onset (witnessed) or within 24 h of having been observed alive and symptom free (unwitnessed)	Multiple source surveillance of all residents of Multnomah County. Oregon	53 per 1,00,000	5.6% of total mortality.
de Vreede-Swagemakers et al^10^ JACC 1997	Sudden cardiac arrest was defined as an unexpected, nontraumatic loss of vital signs, such as consciousness, arterial pulse, BP and respiration, without preceding complaints or within 24 h of the onset of complaints	All victims of unexpected out of hospital SCA between 20 and 75yrs In Maastrict Region.	129 SCAs per 132,762 or 9.7 per 10,000.	18.5% of total mortality.

### 2.2 Indian context

Indigenous data are crucial in guiding clinical protocols and allocating national healthcare resources. However, collecting accurate SCD data in India is challenging due to unique systemic limitations. Major obstacles include under-registration of deaths, unreliable death certificates, often lacking consistent physician attribution and standardized coding, limited emergency services and scarce autopsy data, especially in rural and non-medico-legal cases and sparse outpatient records. These challenges limit the effectiveness of traditional epidemiological tools in India. A verbal autopsy study using pre-designed questionnaires across 45 villages in Andhra Pradesh (population 180,000) identified cardiovascular disease as the leading cause of death, with retrospective analysis revealing SCD accounted for nearly half of these.^12^ Rao et al employed a custom verbal autopsy in the same region, training medical students to administer a two-tiered questionnaire to relatives (covering 22,724 people), with deaths adjudicated by a three-physician panel; key findings included SCD comprising 10.3% of total deaths (mean victim age 61 years; 47% < 60 years, 21% < 50 years; 23% housewives; only 43.4% with prior myocardial infarction(MI)/left ventricular(LV)dysfunction/aborted SCD; 80% with ≥1 coronary risk factor across ages).^13^ An autopsy study from Manipur similarly reported 8.6% sudden deaths, 55% cardiac—mostly due to coronary artery disease (CAD).^14^ These results suggest undiagnosed CAD as a leading cause of SCD in India, particularly among the young. MI and post MI LV dysfunction are established risk factors for SCD.

During the early phase after MI (arrhythmias, recurrent ischemia and cardiac rupture) and during the later phase (myocardial remodeling and macro-reentrant ventricular tachycardia) are important causes, with the first month post–MI being particularly high-risk. The fact that even presumed arrhythmic deaths may be misclassified is reinforced by the differences in results of DANAMI trial (which showed that 63% of cardiac deaths, all presumably arrhythmic, occurred within the first month) and VALIANT trial in which autopsies demonstrated 12% were actually due to cardiac rupture.^15^^,^^16^ While implantable cardioverter-defibrillators (ICD) reduce long-term arrhythmic deaths in patients with remote MI and low LV ejection fraction (EF), they do not benefit early post–MI cases and should not be implanted within 40 days post MI. Furthermore, LV dysfunction alone is insufficient for risk stratification in the early post–MI period.

Post–MI mortality and SCD rates have declined in developed nations due to improved ICU care, timely reperfusion/revascularization, and greater use of guideline-directed medical therapy (GDMT). Indian data, however, shows no such progress: the CREATE registry (20,000+ Acute coronary syndrome (ACS) patients across 89 hospitals; 60% STEMI, mean age 57.5 years) reported delayed presentation (mean ∼6 h), low thrombolysis (58%) and PCI (8%) rates, and high 30-day mortality (9% STEMI, 3.6% non-STEMI).^17^ A single-centre study of 939 STEMI patients over 41 months found half of deaths sudden (mostly first month), with cumulative SCD at 4.9% (1 month), 6.5% (1 year), and 8.9% (3 years)—many pre-ICD eligibility, unlike VALIANT where only 25% occurred early.^18^

**Key Takeaways:**

• Global SCD epidemiology is incomplete, especially in underrepresented regions. Innovative surveillance methods like community-based verbal autopsies and targeted surveys must be further developed and validated to generate actionable Indian data.
• Geographical variation exists in SCD burden and risk factors.
• Developing countries are witnessing a surge in subclinical CAD, with a growing population vulnerable to SCD.
• Many young, economically productive Indians succumb to SCD—often from undiagnosed CAD.
• Primary and secondary prevention, better emergency response, timely ACS care, and urgent focus on early recognition of out of hospital SCA and timely CPR is warranted

## 3 Early recognition of SCA by bystanders and the need for a rapid response

Survival from SCA hinges on the chain of survival (Fig. 1): (1) immediate recognition and emergency activation; (2) high-quality CPR (including dispatcher-assisted); (3) rapid defibrillation; (4) advanced resuscitation; (5) post-arrest care; and (6) recovery/rehabilitation. The first three links—emergency activation, CPR, and defibrillation—offer the greatest survival impact by reaching most patients, yet research funding disproportionately targets advanced care and hospital interventions that benefit fewer cases. Layperson CPR, younger age, shorter emergency medical services (EMS) times, witnessed arrest, public location, shockable rhythms (ventricular fibrillation/pulseless ventricular tachycardia), fewer comorbidities are key survival factors. However majority of SCA events occur at home which complicates the response. Bystander CPR involves four critical steps: (1) recognize cardiac arrest by checking responsiveness, breathing, and carotid pulse within 10 s; (2) call EMS (dial 108 in India); (3) follow dispatcher instructions; (4) start CPR and continue until help arrives, ideally using an AED if available. Dispatchers may guide bystanders to the AED location beforehand.

**Fig. 1:**
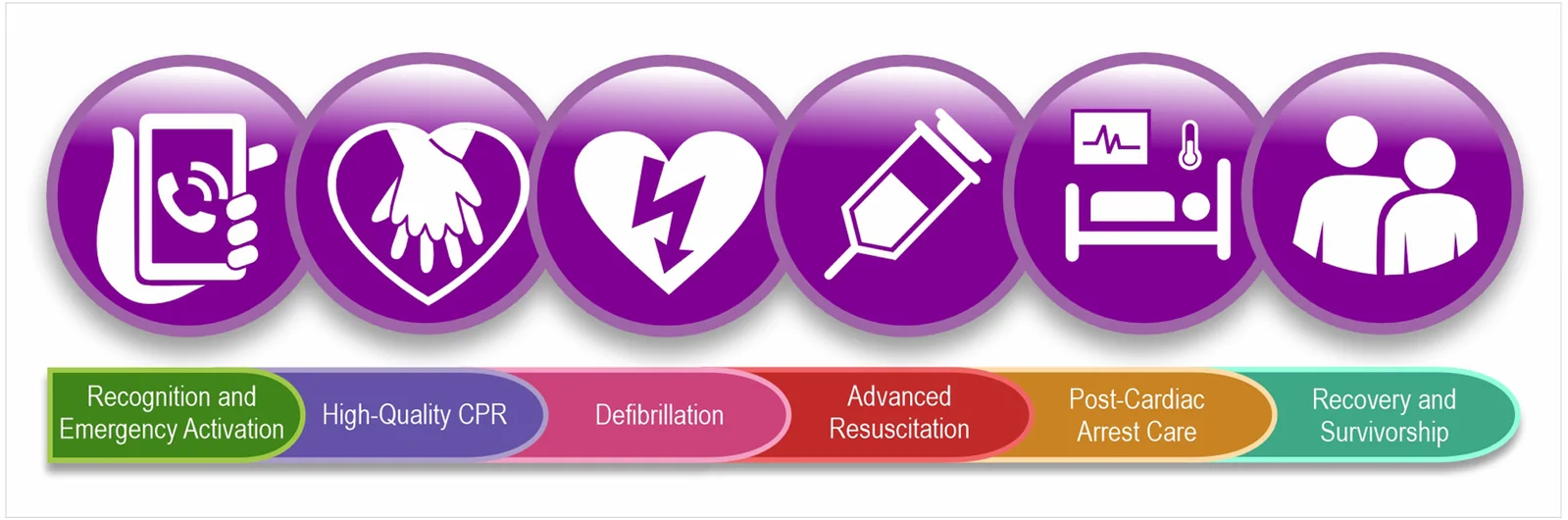
Chain of survival.

### 3.1 Barriers to action

Key barriers include failure to recognize SCA, bystander effect, lack of skills/confidence, fear of harm/legal issues, and aversion to mouth-to-mouth. India's 2016 Good Samaritan Law protects voluntary helpers from liability, allows anonymity, and prevents forced testimony, empowering action. Awareness of this law can empower the public to act without fear of legal entanglement.

### 3.2 Facilitators for uptake

AHA promotes hands-only CPR (no breaths), since 2008 to ease fears of performing CPR incorrectly and infection from mouth to mouth contact, use of brief instructional videos, incorporation of CPR training in to school curriculum, and dispatcher-assisted CPR (DA-CPR).^19^

### 3.3 High-quality CPR

Key elements of effective CPR include (Fig. 2)-

• Chest compression depth: at least 2 inches (5 cm), but not exceeding 2.4 inches (6 cm). Chest compressions should be given with the heel of one hand on the lower half of the sternum, and the heel of the other hand on top of the first so that the hands are overlapped.
• CPR should be performed on a firm surface, if possible without delay, with the victim lying flat (supine) at a rate of 100–120 compressions per minute
• Location: lower half of the sternum
• Recoil: allow the chest to rise fully between compressions
• Performing CPR takes precedence over everything including pulse check. Ideally, no more than 10 s during pulse checks. Check pulse every 2 min and if no pulse, start CPR.^20^

**Fig. 2:**
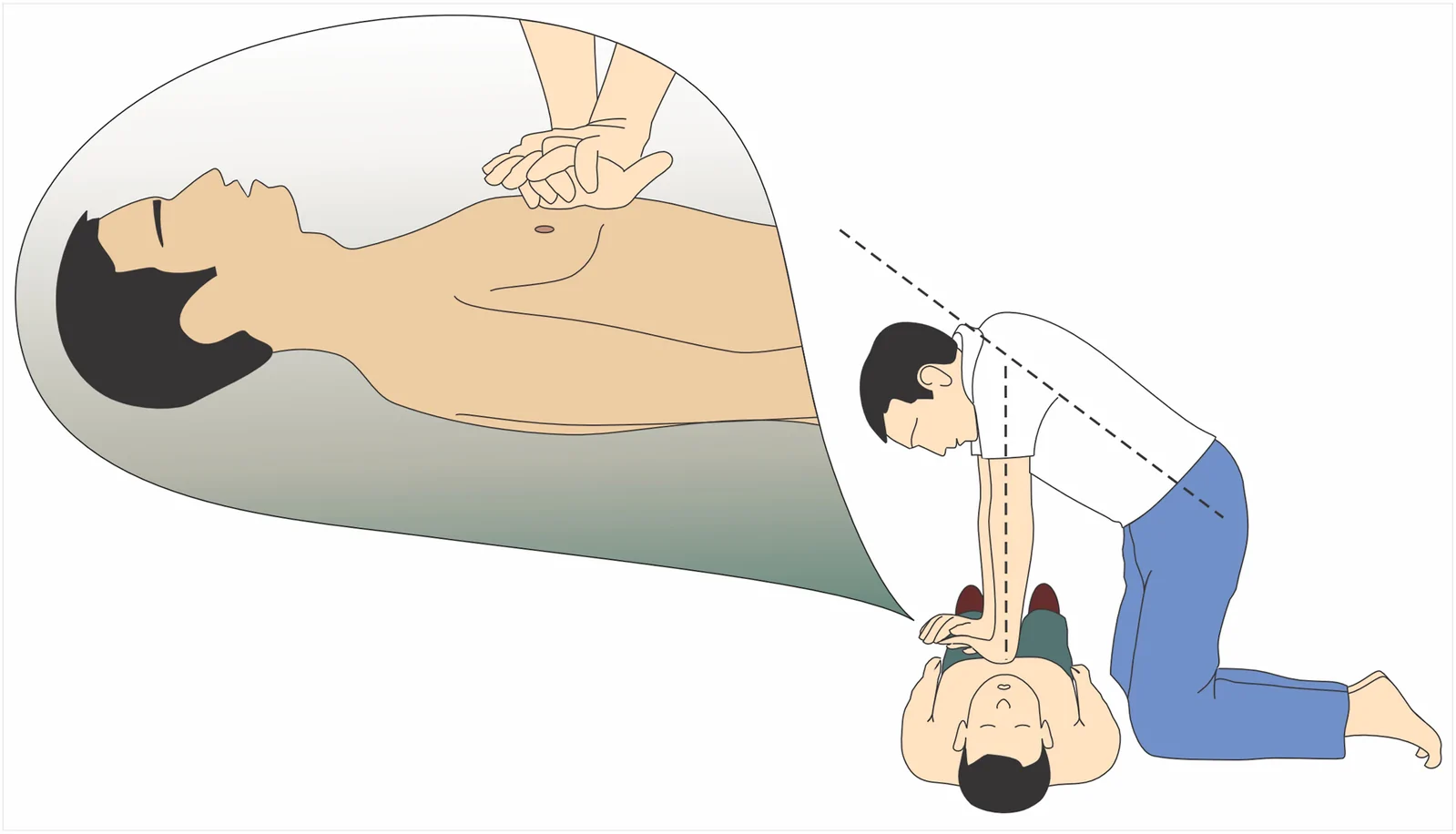
Key elements of effective CPR.

During rhythm checks for adult cardiac arrest, minimize the time taken to check for a pulse (no more than 10 s), and if the rescuer does not definitely feel a pulse, chest compressions should be restarted immediately. (Class of recommendation 1, LOE C-LD).^20^

• The ERC/AHA treatment Recommendations (2025) suggest pausing chest compressions every 2 min to assess the cardiac rhythm (weak recommendation, low-certainty evidence).^21^
• Rotation of rescuers every 2 min when multiple people are present. Switch chest compressors approximately every 2 min (or after about 5 cycles of compressions and ventilation at a ratio of 30:2, (Class of recommendation 2A, LOE B-R).^20^

Even brief interruptions in chest compressions reduce the chance of survival. Early rhythm analysis and defibrillation should be integrated without significant delay in compressions. The 2025 AHA guidelines emphasize the importance of early initiation by stating that resuscitation should generally be conducted where the person is found, as long as high-quality CPR can be administered safely and effectively. However, it is preferred to perform CPR on a firm surface and with the person in the supine position, when feasible and not delay chest compressions.^20^

### 3.4 Dispatcher/video-guided CPR

Dispatchers play a critical role in the early recognition and management of OHCA. Most cardiac arrests are first identified during emergency calls, making prompt and accurate dispatcher recognition essential for initiating timely bystander CPR and appropriate EMS response. There is a wide variability in accuracy of recognition, with the main challenge being the assessment of abnormal breathing and consciousness.^22^^,^^23^ A standard two-question approach (“Is the person conscious?” and “Are they breathing normally?”), is useful enough to improve survival and neurological outcomes irrespective of the language used as an instructional method. The use of simple, direct language reduces time to first compression and improves CPR performance. Therefore, current guidelines strongly recommend standardized dispatcher algorithms and the provision of CPR instructions for all suspected cardiac arrest cases.

Traditionally, dispatchers provide audio-only CPR instructions, but emerging technology now enables video-assisted guidance via mobile phones. Evidence suggests that video instructions improve chest compression fraction and rate, better hand placement at the cost of a slight delay in CPR initiation and no clear benefit in compression depth.^24^ Observational data have shown higher survival to discharge and better neurological outcomes with video-assisted CPR but currently overall evidence is insufficient to recommend widespread implementation.^25^^,^^26^

**Key Takeaways:**

Early recognition of SCA and immediate bystander response is vital and public education should emphasize, (i) immediate Hands-only CPR (ii) Use of AEDs (iii) Legal protection under the Good Samaritan Law.

Technological innovations like dispatcher-assisted and video-guided CPR, optimized AED deployment, and drone-based AED delivery provide promising avenues to improve SCA outcomes. However, at the heart of all this lies a simple truth: an informed and empowered bystander can save a life. Bridging the gap between collapse and care is not just a medical priority—it is a societal responsibility.

## 4 Community based CPR: global and national trends, misconceptions and solutions

### 4.1 Global and national trends

Nearly 70% of OHCA incidents occur at home, 90% of these victims do not survive, and yet only less than half receive immediate help. Since response time is critical, the onus to act lies on the witnessing community members. Global CPR training rates vary widely, depending on public policy and awareness.

Community-based campaigns and initiatives play a vital role in increasing bystander CPR rates and improving response to out-of-hospital cardiac arrest (OHCA). Community CPR training programs, mass-media campaigns, and interventions combining education, policy, and legislative strategies significantly improve bystander response.^27^ Major global initiatives such as *European Restart a Heart Day* and *World Restart a Heart (WRAH)* have demonstrated remarkable impact, training over 12.6 million people and reaching more than 570 million worldwide between 2018 and 2023. These adaptable, culturally sensitive campaigns highlight the importance of coordinated national and international efforts to save lives. The European countries have wide variation in the implementation of CPR. Some European countries including Belgium, Denmark, France, Italy, Portugal, and the United Kingdom have legislation mandating CPR education for school children or prior to obtaining a driver's license. In contrast, many countries recommend CPR training without legal mandates, including Germany, Spain, Sweden, Finland, Poland, Romania, Greece, Turkey, and several others.^28^ Insufficiently trained teachers, limited funding, and regional disparities hinder implementation even where CPR education is mandated.

### 4.2 The Indian scenario

CPR training rates in India are less than 5% but recent initiatives by the Indian Resuscitation Council (IRC), Indian Medical Association (IMA), Cardiological Society of India (CSI), Indian Heart Rhythm Society (IHRS), Red Cross, Heart Rescue India, Revive Heart Foundation, and others are likely to improve these rates. The Ministry of Health and Family Welfare in collaboration with State health departments, central institutions including All India Institute of Medical Sciences, New Delhi, and the Indian Red Cross Society organised a weeklong campaign from 13 to 17 October 2025, to improve survival from SCA. States like Kerala, Tamil Nadu, Karnataka, Maharashtra, Gujarat, and Andhra Pradesh and the Revive Heart Foundation are also advancing community CPR efforts by organizing annual SCA awareness weeks across the country with special focus on educational institutions and large government organisations.

### 4.3 Key challenges in community CPR training

Key challenges like lack of skill/confidence, fear of legal repercussions, criticism from bystanders and reluctance to touch strangers can be solved by training for compression-only CPR, providing visual/audio revision tools, organizing public campaigns, educating about the Good Samaritan Law and sharing success stories. Limited access to training in remote rural/tribal areas can be bypassed by partnering with local groups and delivering training in local languages using radio, TV, or community sessions. Leveraging CSR funds can provide funding support for all such campaigns. Emphasizing scene safety and proper posture can further reduce the risk of rescuer injury, helping to build confidence and promote timely action.

#### 4.3.1 Gender bias in bystander CPR

Evidence shows that women in cardiac arrest are less likely to receive bystander CPR and defibrillation, often due to concerns about chest exposure and fear of inappropriate contact. Women are less likely to receive bystander CPR.^29^ Studies indicate that bras, including those with underwire, do not interfere with defibrillation or cause harm, though delays may occur due to hesitation in exposure.^30^ Current guidance recommends prioritizing rapid pad placement on bare skin in the correct position, adjusting or displacing the bra only if necessary, and not delaying life-saving care for modesty concerns.^31^ Realistic training programs, including female manikins (e.g., Womanikin) can address these barriers.

#### 4.3.2 Compression-Only Life Support (COLS) in India

Introduced in 2017 as a practise guideline by the Resuscitation council formed by the Indian Society of Anaesthesiologists,^32^ the COLS strategy recognizes the fact that there is no consistent superiority of standard CPR over COLS in terms of survival neurological outcomes.^33^ It is suited for laypersons who are not keen to or not trained to provide rescue breaths. It emphasizes early recognition and emergency activation, immediate chest compressions (100–120/min, 5–6 cm depth), and early transfer to medical care. It can be taught telephonically and is ideally adapted to India's cultural and infrastructure challenges. Safety first needs to be emphasized always, and the provider must assess for fire, electrocution, or violence before beginning CPR. Based on current evidence, ERC/AHA recommends that all adults in cardiac arrest receive chest compressions, with trained bystanders providing 30:2 CPR, while untrained or unwilling rescuers perform compression-only CPR.^21^ However it should be noted that these guidelines accord this as only a weak recommendation based on very low–certainty evidence and state that future studies should focus on the benefit of higher compression-ventilation ratios, compared with the current recommendation of 30:2. The latest 2025 AHA guidelines for cardiopulmonary resuscitation and emergency cardiovascular care also give a Class 2a, level of evidence B-R to a compression-ventilation ratio of 30:2.^20^ Healthcare providers may use either approach initially, transitioning to continuous, level of evidence B-compressions with asynchronous ventilation once an advanced airway is secured.

### 4.4 Strategies for implementation of CPR in the community

Community participation requires a culture of action through the principle “Each one teach one.” Key strategies should include regular free CPR training and develop a national CPR initiative by engaging the Ministry of Health and local medical bodies. These training should include police, fire personnel, bus/rail staff, and rural health workers (e.g., Asha, Anganwadi staff). CPR training kiosks should be installed at airports, stations, religious sites. Integration of CPR training into school and college curriculum, use of technology: videos, apps, advanced mannequins, multilingual television/radio advertisements and social media content, encouragement of 108- call operators to guide CPR remotely should be done. Given India's vast rural population and emergency care challenges, widespread CPR training is critical. Community-based programs can empower bystanders to act swiftly and save lives. Aligning these efforts with national policies and public campaigns will significantly improve survival outcomes from SCA.

**Key takeaways:**

Given India's vast rural population and emergency care challenges, widespread CPR training is critical. Community-based programs can train bystanders to act swiftly and save lives. Aligning these efforts with national policies and public teaching campaigns will significantly improve survival outcomes from SCA.

## 5 Public Access Defibrillation (PAD)

Delays in EMS response times make bystander use of PAD critical. The AHA has defined PAD as making an AED available in public and/or private places where large numbers of people gather or where people who are high risk for heart attacks reside. Survival rates with AED's depends on whether the arrest is witnessed or not, is shock is delivered immediately and system performance since most OHCAs happen in private homes, where AED access is limited.

### 5.1 Evidence favoring PAD

The PAD trial, a prospective, community-based study reported that with laypersons trained in both CPR and AED use, survival rates doubled, without delivering any inappropriate shocks.^34^ Other studies have also reported that integrating AED training into an organized CPR response system can improve survival rates from OHCA, without increasing neurological complications.^35^^,^^36^ Every minute of delay in defibrillation reduces survival by 9%, making rapid AED use crucial.

### 5.2 AED placement and accessibility

AED placement should balance event likelihood and community impact and there is a growing public willingness to use AEDs when appropriately placed and maintained. Previous AHA guidelines suggested that AEDs be placed where the frequency of cardiac arrest events is such that there is a reasonable probability of one AED use in 5 years (estimated event rate of 1 sudden cardiac arrest per 1000 person-years).^37^ Subsequent studies recommend two AEDs and at least 10 volunteer first responders per km^2^ for early access to resuscitation resources.^38^^,^^39^ For pedestrian-based access, AEDs should ideally be within 100 m of an OHCA allowing for defibrillation within the 3-min timeframe recommended by AHA.^40^ However, such numerical metrics may not carry too much significance without linking them to geographic distribution of OHCA. AEDs must be placed where SCAs are most likely to occur.

### 5.3 Positioning of defibrillation pads

There is no evidence that pad size affects return of spontaneous circulation (ROSC), survival, or neurological outcome. Studies comparing anterior-posterior and anterior-lateral placement found no significant differences in survival, neurological outcome, or defibrillation success, although adjusted analyses suggested slightly higher ROSC with anterior-posterior positioning. Overall, there is insufficient evidence to recommend a specific pad size or position. An anterior-lateral placement is the usual approach because it is quicker and minimizes interruptions to chest compressions.^41^ In this position, one pad is placed below the right clavicle beside the sternum and the other on the left mid-axillary line below the armpit. An anterior-posterior position (chest and back) may be used if anterior-lateral placement is not feasible and the rescuer is trained. Pads must be placed at least one inch(2.5 to 3 cm) away from the device site in patients with implanted pacemaker or ICD (Fig. 3). Women in cardiac arrest are less likely to receive CPR and defibrillation, partly due to reluctance to expose the chest. Pads must be applied to bare skin in the correct position; this can often be achieved by adjusting rather than removing the bra. If the garment interferes with proper pad placement it should be displaced, and rescuers should prioritize life-saving care over concerns of modesty. Training programs should include realistic scenarios involving pad placement in patients wearing bras.

**Fig. 3:**
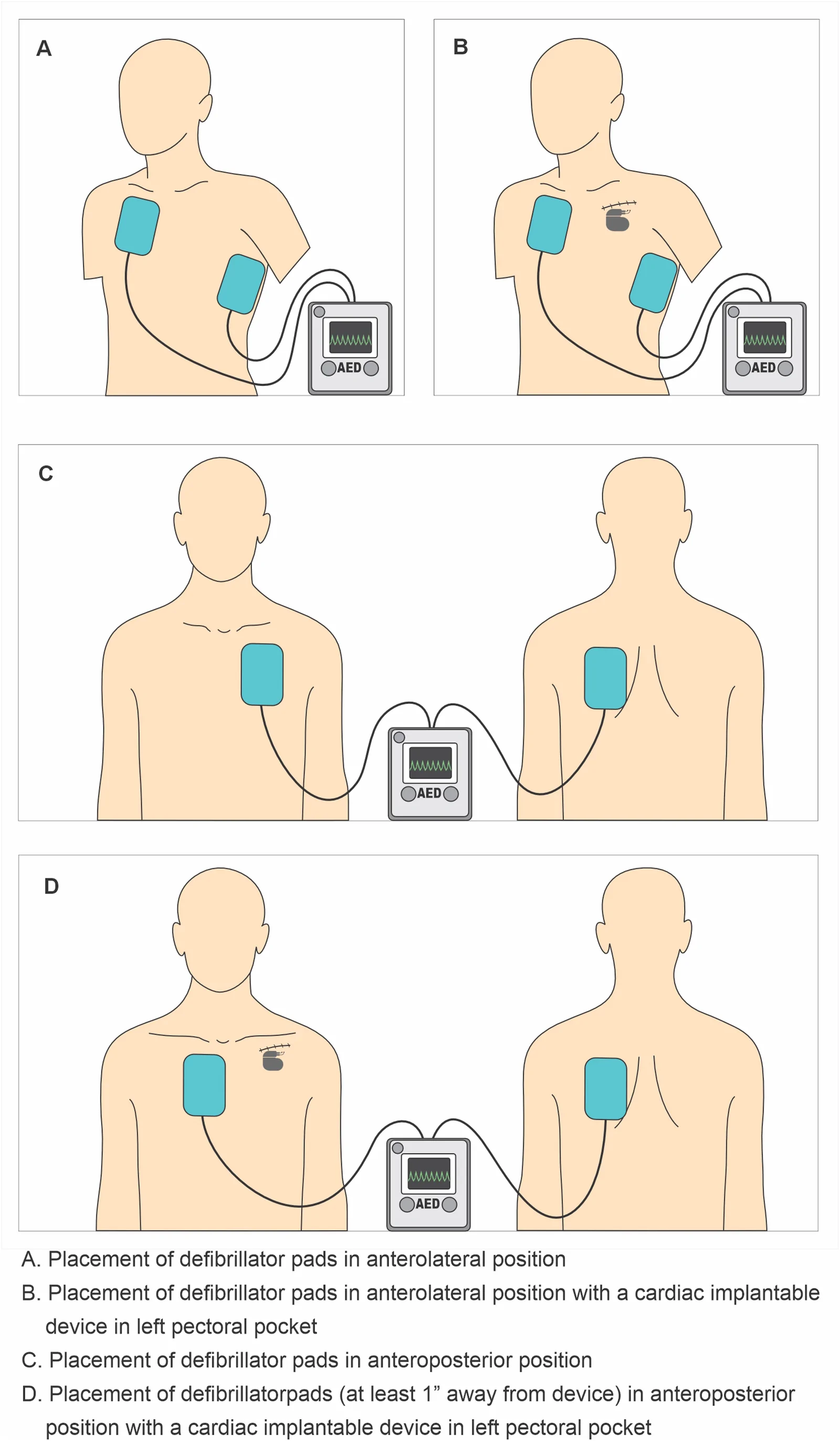
Positioning of defibrillation pads.

**Fig. 4:**
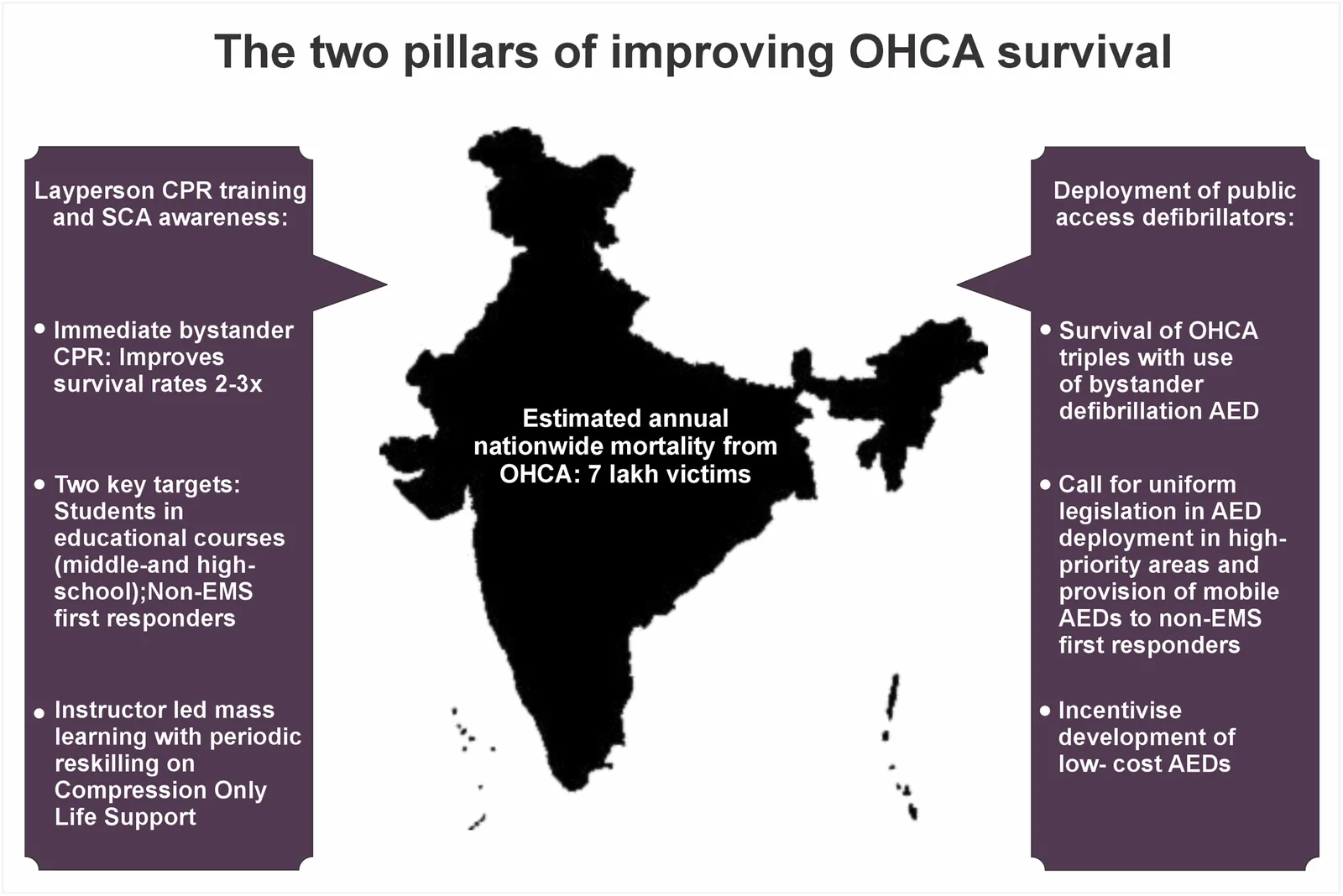
Areas needing legislative focus.

### 5.4 Challenges in PAD

Deployment of PADs without central coordination, oversight or accountability, is unlikely to be cost-effective. Many are installed where few arrests occur, and most are inaccessible outside business hours. Though PAD is safe, with no inappropriate shocks reported in large trials, using an AED and performing CPR can be a challenge for laypersons. Despite training, only 50% of volunteers actually performed CPR and only 7% delivered shocks. Reported issues included stolen AEDs and stress among rescuers.^42^ Furthermore, the costs associated with purchase, maintenance, and training, poor visibility, lack of signage, locked cabinets, and fear of legal consequences remain key barriers to widespread and effective PAD implementation in India.

### 5.5 Expanding PAD strategies

AED placement at large crowd locations such as airports, railway stations, bus stations, schools shopping malls, sports venues, and religious places like temples, mosques and churches can significantly improve survival. Geolocation-enabled mobile applications with national AED registries can help direct bystanders and responders to both the patient and the nearest available AED; although the trial failed to show increase in AED use when responders were guided to AEDs en route.^43^ Smart AED stations and connected systems including wearables and contactless devices capable of detecting agonal breathing and automatically alerting EMS, while web-enabled AEDs can report location and maintenance status and provide visual and audio cues to guide bystanders—features particularly suited to semi-public settings such as residential complexes. Singapore's “AED-on-wheels” initiative involving taxi drivers, and by equipping police, firefighters, and off-duty medical personnel, may be relevant for India, where AED availability remains limited. Additional innovations include drone-delivered AEDs for rapid access in congested, remote, or rural areas and at mass gatherings, as well as emerging compact and affordable have the potential to broaden access and enable earlier defibrillation.

**Key takeaways:**

PAD significantly improves survival in public, witnessed SCAs. Innovations in technology, better AED placement, and integrated EMS-volunteer systems can improve access to PAD. Authorities must prioritize funding and infrastructure to increase AED visibility, accessibility, and public readiness. A paradigm shift toward home-based and mobile AEDs may be essential to truly impact survival rates across the OHCA spectrum. Even with increased AED availability, PAD programs will remain suboptimal without widespread BCLS training to ensure effective use.

## 6 Improving CPR success in society: CSR initiatives for AED installation

AED installation in public places is rare in India, even in major cities with advanced infrastructure. High-risk locations such as stadiums, parks, railway stations, and schools inadequately equipped. Lack of awareness, funding challenges, unclear ownership and absence of policy mandates are the main reasons. Although governments, non-government organisations (NGO), and public health bodies have roles to play, there is one stakeholder uniquely positioned to make an immediate and measurable difference: the corporate sector. In the absence of any robust government policy, CSR initiative offer a powerful, underutilized means not just for deploying AEDs, but also for increasing public awareness and building community-level cardiac emergency response systems. Role of CSR in India is well defined under Clause 135 of the Companies Act, 2013. Companies meeting specified financial thresholds (net worth of ₹500 crore, turnover of ₹1000 crore, and/or a net profit of ₹5 crore or more) must spend 2% of their net profits on CSR.^44^ While most CSR funds go to education, sanitation, or rural development, the Act also permits healthcare support—including emergency preparedness like AEDs. Corporate sector can earn public goodwill while empowering communities. AEDs are low-maintenance and scalable. It will directly benefit the working-age population, as most sudden cardiac arrest victims in India are in their 40s or 50s. Despite successfully ticking most of these boxes, large-scale AED deployment under CSR in India remains limited. Costs, accountability, and lack of public knowledge do pose challenges. Yet with proper planning and intent, these are solvable.

Global and local experiences demonstrate feasibility and impact. Countries like Singapore and corporations such as American Airlines and TESCO have successfully implemented large-scale AED programs,^45^, ^46^, ^47^ while in India, initiatives by the Rotary Club of Bombay Airport and the Revive Heart Foundation have already resulted in lives saved through strategic AED placement. Similar CSR-supported efforts in cities such as Pune, Dehradun, Lucknow, and Nagpur highlight growing momentum, but these remain fragmented and lack coordination. What is needed is a scalable, city-wide model, such as a seven-point CSR blueprint exemplified by Mission “Dil Dhadakne Do” in Jamshedpur, encompassing stakeholder collaboration, public awareness campaigns, robust AED infrastructure, structured training of first responders, technology integration through AED-mapping and CPR-guidance apps like REVIVE-CPR that was launched on 14th December 2024, continuous monitoring of outcomes, and sustained policy advocacy. Cardiology community can help guide the CSR investments by ensuring high-quality CPR training and overseeing the long-term sustainability of AED programs. National professional bodies such as the Cardiological Society of India (CSI), Indian Heart Rhythm Society (IHRS) and Indian Society of Electrocardiology (ISE) can further amplify impact by drafting standardized AED deployment guidelines, advocating mandatory CPR training in schools, and supporting the development of a national AED network integrated with emergency medical services.

**Key takeaways:**

Although India has the resources and CSR framework to act, it needs to be emphasized that corporate social responsibility is a potential funding mechanism, not a substitute for public health infrastructure. We need to move from scattered efforts to structured strategy, from reaction to readiness, and from awareness to action.

## 7 CPR training in school and college curriculum

Integration of CPR training in the school curriculum can significantly increase the SCA survival rates. It can make a transformative public health impact with more than 250 million students across the country by making them competent in handling cardiac emergencies. The National Education Policy (NEP) 2020 has strong emphasis on life skills, experiential learning, and holistic education which provides an opportunity for inclusion of CPR training. Focusing on children and young adults offers clear advantages: over half of India's population is under 25 years of age,^48^ young learners are particularly receptive to acquiring cognitive and motor skills such as CPR, and students often act as social multipliers by sharing their knowledge with families and communities, thereby amplifying awareness and preparedness.

Global experience strongly supports early CPR education. Highest bystander CPR rates are reported from countries such as Norway, Denmark, Singapore, and the United States which have made CPR training mandatory in schools, with 43 US states requiring CPR instruction as a prerequisite for high school graduation.^49^ The “Kids Save Lives” initiative advocates for at least 2 h of annual CPR training starting at age 12 while empowering children to recognize cardiac arrest, call emergency services, perform chest compressions, and raise awareness within their communities.^50^^,^^51^ The WeCan CPR training program in China has also introduced compulsory CPR education in high schools, universities and colleges.

**The Indian context**: Despite sporadic NGO-led efforts, India still lacks standardized, large-scale CPR training in educational institutions. A 2019 bill proposing compulsory CPR education in schools remains unenacted, but existing platforms such as NEP 2020 and national initiatives like the NBEMS Nationwide CPR Awareness program provide a strong foundation for reform. A structured roadmap for integration would involve coordinated policy advocacy with the Ministries of Health and Education, alignment with NEP goals, and mandates from education boards such as CBSE, ICSE, State Boards, and UGC. Curriculum development should be undertaken in collaboration with organisations like the Indian Resuscitation Council, Red Cross, and St. John Ambulance, with age-appropriate modules ranging from emergency recognition in middle school to hands-only CPR in high school and certified CPR and AED training at the college level. Instructor capacity can be built through a train-the-trainer model involving teachers, physical education instructors, and healthcare professionals, supported by national cardiology and resuscitation bodies. Implementation can be achieved through annual CPR awareness weeks, student-led CPR clubs in partnership with NCC and Scouts, and digital learning platforms to ensure reach in rural areas. Assessment, certification, and continuous monitoring should be embedded into the system, supported by government funding and augmented by CSR and philanthropic contributions. While challenges such as trainer shortages, infrastructure gaps, and time constraints exist, these can be effectively addressed through low-cost training tools, regional workshops, and integration of CPR training into existing school schedules, making CPR education both feasible and sustainable at a national scale.

**Refresher Training Intervals:** CPR knowledge and psychomotor skills decline significantly within months after initial training. Therefore, short, frequent refresher sessions for school children every 6–12 months using video-based modules, simulation drills, or app-guided practice can improve long-term retention, confidence, and bystander response rates. **School- and workplace-based retraining programs, teacher training and low-cost manikin practice** may provide sustainable large-scale reinforcement in India. Training school teachers, community health workers, and local volunteers as CPR instructors can help expand outreach in resource-limited settings.

**Key takeaways:**

If implemented systematically, CPR education in schools and colleges, can substantially raise India's bystander CPR rates closer to global standards and move India toward a fully CPR-literate society by 2047.

## 8 Legislation and policy advocacy for SCA and cardiopulmonary resuscitation awareness in India

Strong legislative backing and coordinated policy advocacy is required for improving survival in SCA in India. The “chain of survival” concept assumes equal feasibility of each step, an assumption often unrealistic in resource limited settings.^52^ Recently ILCOR proposed the “chainmail of survival,” which acknowledges variability across systems and highlights areas where legislation and policy support are essential. Complementing this, the Utstein “Formula for Survival”—includes interaction of scientific evidence, education and training, and local implementation—offers an objective framework to assess gaps in care and target system-level improvements.^53^ Recently Ministry of Health and Family welfare (Government of India) organised a CPR awareness week (13th −17th October 2025) to improve public readiness for compression only CPR and bystander response during cardiac arrest. It engaged 7.47 lakh citizens and over 6 lakh individuals received CPR training through hands-on training, demonstrations and digital outreach. India lacks a national OHCA registry, hampering understanding of SCD's epidemiological burden. For perspective, China, with ∼1.3 million annual OHCA cases and <2% survival, legislated public BLS training, AED deployment, and Good Samaritan protections via the Emergency Medical Aid Act, passed in 2018.^54^ This has resulted in significantly higher bystander CPR and AED use, and improved survival in pilot cities that have implemented the act.

### 8.1 Areas needing legislative focus are listed below of which high-priority attention should be on the first two areas (Fig. 4)

1. PADs
2. Layperson CPR training
3. Nationwide OHCA Registry
4. Good Samaritan law
5. Telecommunicator CPR (T-CPR)
6. Rapid response system access
7. Bystander notification via Mobile dispatch

Defibrillation within 3–7 min improves OHCA survival dramatically. Compared to E MS-only programs, PAD deployment significantly improves Return of Spontaneous Circulation (ROSC) and survival outcomes. Although AHA recommends PADs in high-risk areas, India faces primarily three challenges –

1. High cost & non-uniform availability: Only a few cities mandate AEDs in government

buildings. Central legislation must mandate PADs in high-traffic private and public venues, with defined installation and maintenance protocols. A “Make in India” initiative for low-cost AEDs should be prioritized.

2. Training for AED Use
3. Limited home access: Most SCAs occur at home. Immediate solutions include training non- EMS first responders (police/firefighters) in AED use and equipping them with local AED access information. Volunteer responder programs and AED drones are emerging but costly for widespread use in India. Layperson CPR training can include school and college students, non-EMS first responders (especially police), staff in high-risk community areas (schools, offices), family members of cardiac patients. Blended training (instructor-led + self-directed) with feedback is superior. COLS has shown better outcomes for laypersons and the IRC's 2017 COLS guidelines provide a good roadmap for this.^32^ “Mass learning” sessions should be followed by periodic booster training (monthly or 6-monthly) to reinforce skills. Given current constraints in personnel, resources, and awareness, layperson CPR training is arguably the most critical national priority. Presently, training is fragmented, left to local NGOs and institutions. A central effort under the National Health Mission is urgently needed. A national registry would clarify India's burden of OHCA, and the operational elements could include standardized national data collection formats, Integration with EMS, hospitals, police, and mortuary data, patient identification and real-time digital reporting platform, geographic mapping of OHCA hotspots, data on bystander CPR and AED use, outcome monitoring and quality indicators and benchmarking systems.

. Although some institutional registries exist, national-level mandatory reporting is essential. India's 2016 Supreme Court Good Samaritan Law protects bystanders offering emergency help. However, public awareness is minimal, and fear of legal complications continues to deter action during SCAs. Dissemination of information to the public regarding this legislation remains limited forming a socio-cultural barrier that prevents bystanders from actively assisting SCA victims. T-CPR enables dispatchers to guide callers through CPR while dispatching EMS. This requires training telecommunicators, ensuring ongoing quality checks, and coordination with EMS. AHA mandates EMS dispatch within 90 s of call initiation—a benchmark India must aim for. There has been significant effort and improvement in EMS response time over the prior two decades in our country. However, EMS response times continue to be longer (up to 20 min) in many states, especially in remote and conflict-prone areas. The Emergency Response Support System (ERSS) with real-time tracking and a single helpline promises nationwide improvement, contingent on public awareness.

Smartphone apps to alert trained bystanders of nearby OHCA incidents have shown promise abroad.

India should evaluate feasibility through pilot projects. Policies to improve healthcare literacy and awareness utilizing information broadcasting including conventional and social media campaigns would increase the impact of legislative interventions.

**Key takeaways:**

Mandatory legislation for AED placement in public spaces is critical to improve survival from sudden cardiac arrest (SCA). First responders including police and fire services should be equipped and trained to use AEDs. Promotion of low-cost, indigenous AED manufacturing will enhance accessibility and scalability across India and integration of CPR and AED training into public health policy can empower laypersons to act during emergencies.

## 9 Use of innovative technologies and online resources for CPR training

Learning CPR is not a one-time event; it requires regular revision and skill refinement. To ensure ongoing engagement and expand outreach, innovative technologies and digital strategies must be leveraged. In today's internet-savvy society, tools like virtual simulations, mobile apps, and social media offer scalable and accessible solutions to CPR education and SCA awareness.

### 9.1 Digital strategies in CPR education

Digital technologies such as mobile apps, wearable devices, AI, visual media, and crowdsourcing are transforming CPR education and sudden cardiac arrest (SCA) response. These tools provide instant, personalized learning in local languages through videos, simulations, feedback systems, and real-time guidance. Emergency services can also benefit from GPS tracking, photo/video sharing, and rapid communication. Social media campaigns and influencers further improve awareness and community participation. The European Restart a Heart Day has now evolved into the global World Restart a Heart (WRAH) campaign endorsed by ILCOR with the message “Check, Call, Compress.” Between 2018 and 2023, WRAH trained over 12.6 million people and reached more than 570 million individuals worldwide.^28^ AI-enabled wearables such as smartwatches, ECG patches, and infrared/radar systems can detect agonal breathing, abnormal rhythms, falls, or inactivity suggestive of impending cardiac arrest. These systems may alert emergency services automatically and also help generate data for improving cardiac arrest research and interventions.

Digital technologies now allow training anytime and anywhere through online videos, virtual simulations, and immersive multilingual environments such as the metaverse. Mobile CPR units equipped with mannequins and AEDs can conduct public training in airports, railway stations, and conferences. Audio prompts, metronomes, video refreshers, and smartwatch-based feedback systems improve compression quality and skill retention.

Websites such as reviveheartfoundation.org and saveheartsfoundation.org provide reliable CPR educational content and training programs. Mobile applications are also important tools for prevention and emergency response. Apps integrated with ECG and heart-rate monitoring systems such as Apple Watch ECG and KardiaMobile can identify high-risk individuals and issue alerts. Others promote healthy lifestyles, medication adherence, smoking cessation, stress management, and sleep improvement.

Apps like PulsePoint (USA), LifeSaver (UK), and Savelife (India) notify nearby trained volunteers during cardiac arrest events, reducing response time. Many apps also provide CPR instructions, metronomic guidance, and GPS-based AED location services. Telemedicine platforms such as those used by Narayana Health have improved outcomes in high-risk cardiac patients.

### 9.2 Social media campaigns and crowdsourcing

Social media has become a powerful tool for spreading CPR awareness through campaigns such as “Hands-Only CPR” using hashtags, videos, blogs, and influencers. Online initiatives like the Defibrillator Design Challenge improved public awareness and visibility of AEDs through community participation and innovative designs. Crowdsourcing projects such as the MyHeartMap Challenge successfully mapped AED locations through public submissions of photographs and locations, creating highly accurate AED registries. Similar approaches can be adapted in Indian cities to improve AED accessibility, raise funds, and organize CPR training in underserved communities.

### 9.3 Challenges and limitations

Despite their promise, digital CPR strategies face several challenges. Many online CPR videos contain inaccurate or incomplete information. Long-term engagement with health apps is often poor, and protection of sensitive health data requires strict compliance with privacy regulations such as General data protection regulation (GDPR). Government health bodies should integrate social media strategies into resuscitation programs, leverage emerging platforms (You tube, LinkedIn), and deploy artificial intelligence (AI) for fact-checking, targeting bystander CPR uptake and SCA survival.^55^^,^^56^

AI has shown encouraging results in rhythm identification, CPR optimization, and survival prediction, but most studies remain retrospective and require prospective validation. Additional concerns include algorithm bias, public trust, language barriers, low digital literacy in rural areas, and integration with healthcare systems and Electronic Health Records (EHRs).^57^ Further research is needed to measure the direct impact of digital CPR tools on survival outcomes.

**Key Takeaways**.

Digital tools including apps, simulations, wearable devices, AI, and social media are reshaping CPR training and SCA prevention. They provide real-time learning, personalized guidance, and faster emergency response. Indian platforms such as Savelife and mCessation are already demonstrating measurable benefits. Integrating these innovations with traditional healthcare systems can strengthen the chain of survival and improve outcomes after sudden cardiac arrest.

## 10 Understanding the psychological impact of SCA and CPR on bystanders: support and interventions

The bystander effect, a socio-psychological phenomenon where individuals are less likely to intervene in an emergency situation when others are present, can have profound implications in cardiac arrest scenarios. Understanding this psychological phenomenon is key to improving outcomes, as hesitation can result in precious seconds lost.

### 10.1 Factors contributing to bystander effect in SCA scenarios

**Pluralistic ignorance**: Bystanders may look to others for cues on how to respond, especially if they are unsure of what to do. If everyone appears calm or hesitant to act, individuals may interpret this as a signal that intervention is unnecessary or inappropriate, even if they recognize the severity of the situation. They may experience **evaluation apprehension**, fearing judgment or scrutiny from others if they intervene. They may worry about making mistakes or causing harm, particularly if they lack confidence in their CPR skills or are unfamiliar with operating an AED. **Social influence** also plays a role in the bystander effect. Bystanders may conform to the behavior of those around them, especially if they perceive others as more knowledgeable or competent. To counteract these obstacles, education and empowerment are critical. Public CPR training, including in schools and workplaces, enhances confidence and readiness. Installing AEDs in public spaces and raising awareness through media campaigns help ensure that people are both willing and able to act. By making the bystanders feel competent and responsible, we can create a culture of bystander intervention where every individual feels empowered to make a difference in an emergency.

### 10.2 Psychological impact on bystanders

Beyond this hesitation lies a deeper emotional burden. Witnessing a cardiac arrest is intensely distressing. Bystanders often report shock, helplessness, fear, and guilt, regardless of whether they intervene. Those who do step in—especially performing CPR—may later question their actions, worry about the outcome, have intrusive thoughts or even relive the moment through flashbacks and nightmares. This can lead to moral distress, anxiety, or symptoms of post-traumatic stress disorder (PTSD).

### 10.3 Impact on families

Families of SCA victims, especially if present during the event, may experience trauma, grief, and fear of recurrence, even if the victim survives. The sudden, life-threatening nature of cardiac arrest can leave families emotionally shattered and uncertain about the future. Psychological support and counselling are essential to help them process what happened, cope with grief or anxiety, and regain a sense of stability.

### 10.4 Support for survivors

Survivors of SCA face a unique psychological journey. Having faced a near-death experience, they are prone to PTSD, anxiety, and depression. Lingering fears about their health, dependency on others, or the meaning of their experience can lead to emotional and existential distress. Specialized mental health support, such as cognitive-behavioral therapy or trauma-informed care, can be life-changing in helping them recover and adjust. Psychologists often play a pivotal role in promoting psychological well-being and enhancing overall quality of life post-cardiac arrest. Importantly, PTSD can affect all groups involved - bystanders, families, and survivors. Common symptoms include re-experiencing the event, emotional numbness, sleep disturbances, and hypervigilance. These responses are natural but can interfere with daily functioning. It is essential to recognize the potential for PTSD among all those affected by SCA and provide appropriate support and interventions to mitigate its impact. A multi-disciplinary approach involving medical, psychological, physical, and occupational support often play a pivotal role in promoting well-being and enhancing overall quality of life post-cardiac arrest.

In the immediate aftermath of a SCA, Psychological First Aid (PFA) can provide essential emotional care. PFA can be provided by mental health professionals, trained responders, or even volunteers with basic skills. Its aim is to reduce distress, foster resilience, and help individuals return to normal functioning after trauma. Cardiac arrest survivor organisations are community based organisations supporting survivors. These organisations focuses on various economic needs, practical assistance, social networks, and legal issues of survivors beyond just healthcare. They also promote research in this field and create survivor communities where individual can share their experiences. Patient and Public involvement and Engagement (PPIE) refers to involvement of people in developing healthcare guidelines and contribution in policy decisions. Engagement of PPIE in resuscitation science is a relatively new concept, but its potential remains largely unexplored and untapped.

**Key takeaways:**

The psychological impact of SCA and CPR transcends the realm of immediate medical emergencies, exerting a profound influence on bystanders, families, and survivors alike. Acknowledging and addressing this emotional impact is vital.

Through the provision of tailored support, education and interventions, psychologists play a pivotal role in ameliorating the enduring psychological effects of SCA and empowering individuals to reclaim agency over their psychological well-being. Encouraging a culture of bystander action and emotional resilience will improve survival and recovery in OHCA scenarios.

## 11 The ten commandments of CPR awareness in India

Survival from SCA depends on immediate bystander action. Lives can be saved with coordinated efforts of society and doctors aimed at strengthening the overall structure, care pathways, and quality of care. Following commandments highlights a pathway to improve SCA outcomes in India.

1. Act immediately – Recognize cardiac arrest quickly and begin CPR without delay; seconds matter.
2. Ensure safety first – Protect yourself and the victim by securing the scene before intervention.
3. Call for help – Activate emergency services early; professional care must be mobilized alongside layperson action.
4. Check responsiveness and breathing – Confirm unresponsiveness and abnormal breathing to avoid hesitation.
5. Deliver high-quality chest compressions – Hard, fast, and uninterrupted compressions are the foundation of effective CPR.
6. Use AEDs promptly – Early defibrillation can reverse fatal arrhythmias; AEDs are designed for anyone to use safely.
7. Promote training in schools and workplaces – Embedding CPR in curricula and corporate programs builds long-term resilience.
8. Dispel myths and misconceptions – Fear of harm, lawsuits, or “doing it wrong” must be replaced with confidence and courage.
9. Leverage technology – Digital apps, VR tools, and online platforms make CPR learning accessible and engaging for all.
10. Join national and global campaigns – Awareness drives such as World Restart a Heart Day inspire mass participation and normalize lifesaving action.

## 12 Conclusion

Improvement in SCA outcomes by increasing awareness of CPR requires a profound societal responsibility. In the event of SCA, it is often not a doctor but a bystander who can make the ultimate difference between life and death. Therefore, widespread public education, effective debunking of prevailing myths, and accessible CPR training programs are essential. To enhance reach and engagement, CPR training initiatives should incorporate innovative strategies, including mobile applications, large-scale social media campaigns, virtual skills-based learning platforms, and community-based mass training events. Consideration should be given to implementing mandatory CPR certification at key societal milestones, such as secondary school graduation, issuance of driving licences, and hospital discharge. Each individual trained in CPR represents a potential lifesaver. By fostering a culture in which timely intervention during a crisis becomes instinctive, we can empower communities and ensure that life-saving actions are truly within everyone's reach.

## Declaration of competing interest

The authors declare that they have no known competing financial interests or personal relationships that could have appeared to influence the work reported in this paper.
